# Phase-amplitude coupling and infraslow (<1 Hz) frequencies in the rat brain: relationship to resting state fMRI

**DOI:** 10.3389/fnint.2014.00041

**Published:** 2014-05-27

**Authors:** Garth J. Thompson, Wen-Ju Pan, Jacob C. W. Billings, Joshua K. Grooms, Sadia Shakil, Dieter Jaeger, Shella D. Keilholz

**Affiliations:** ^1^Magnetic Resonance Imaging of Neural Dynamics Lab, Department of Biomedical Engineering, Georgia Institute of Technology and Emory UniversityAtlanta, GA, USA; ^2^Department of Electrical and Computer Engineering, Georgia Institute of TechnologyAtlanta, GA, USA; ^3^Computational Neuroscience Lab, Department of Biology, Emory UniversityAtlanta, GA, USA

**Keywords:** functional MRI, slow cortical potentials, DC potentials, cross-frequency coupling, nested oscillations, resting state, spontaneous activity, multiscale activity

## Abstract

Resting state functional magnetic resonance imaging (fMRI) can identify network alterations that occur in complex psychiatric diseases and behaviors, but its interpretation is difficult because the neural basis of the infraslow BOLD fluctuations is poorly understood. Previous results link dynamic activity during the resting state to both infraslow frequencies in local field potentials (LFP) (<1 Hz) and band-limited power in higher frequency LFP (>1 Hz). To investigate the relationship between these frequencies, LFPs were recorded from rats under two anesthetics: isoflurane and dexmedetomidine. Signal phases were calculated from low-frequency LFP and compared to signal amplitudes from high-frequency LFP to determine if modulation existed between the two frequency bands (phase-amplitude coupling). Isoflurane showed significant, consistent phase-amplitude coupling at nearly all pairs of frequencies, likely due to the burst-suppression pattern of activity that it induces. However, no consistent phase-amplitude coupling was observed in rats that were anesthetized with dexmedetomidine. fMRI-LFP correlations under isoflurane using high frequency LFP were reduced when the low frequency LFP's influence was accounted for, but not vice-versa, or in any condition under dexmedetomidine. The lack of consistent phase-amplitude coupling under dexmedetomidine and lack of shared variance between high frequency and low frequency LFP as it relates to fMRI suggests that high and low frequency neural electrical signals may contribute differently, possibly even independently, to resting state fMRI. This finding suggests that researchers take care in interpreting the neural basis of resting state fMRI, as multiple dynamic factors in the underlying electrophysiology could be driving any particular observation.

## Introduction

Resting state functional magnetic resonance imaging (fMRI) maps brain networks when no explicit task or stimulation is present using similarities in infraslow-frequency fluctuations in the blood oxygenation level dependent (BOLD) signal. As these networks are found by comparing correlated functional signals from across the brain, they are referred to as a “functional networks” (Biswal et al., 1995). Differences in fMRI-measured functional networks have been linked to many neuropsychiatric diseases (Greicius et al., 2004; Villalobos et al., 2005; Tian et al., 2006; Garrity et al., 2007; Zang et al., 2007; Van Den Heuvel and Hulshoff Pol, 2010) as well as behavior variation in healthy humans (Waites et al., 2005; Hampson et al., 2006; Weissman et al., 2006; Boly et al., 2007; Albert et al., 2009; Thompson et al., 2013c).

Early studies of resting state fMRI examined functional networks as if they were static. However, recent evidence has emerged of at least two types of dynamic changes that can be observed in the resting state BOLD signal. First, the correlative metrics used to calculate functional connectivity need not be calculated over an entire fMRI run. Instead, they can be calculated over segments as short as the period of the low-pass filter to reveal changes in correlation over time (typically the correlation is measured between two brain sites using the same modality, Chang and Glover, 2010; Hutchison et al., 2013b; Keilholz et al., 2013; Thompson et al., 2013a). Second, visual observation of resting state fMRI data reveals that certain large scale patterns repeat themselves (Majeed et al., 2009), suggesting a spatiotemporal organization to the infraslow fluctuations. These spatiotemporal patterns were quasi-periodic (periodic, but not constantly active). The first type of dynamic, sliding window correlation, has been robustly observed in rats, monkeys, and humans (Chang and Glover, 2010; Kiviniemi et al., 2011; Tagliazucchi et al., 2012; Hutchison et al., 2013b; Keilholz et al., 2013; Allen et al., 2014) and may relate to behavior and to disease (Sakoglu et al., 2010; Thompson et al., 2013c). The second type of dynamic, quasi-periodic patterns (QPP), have been detected in rats and humans (Majeed et al., 2009, 2011), and similar patterns have been seen in human subjects using different methods including a triggered averaging algorithm in both BOLD (Majeed et al., 2011) and cerebral blood volume imaging (Magnuson et al., 2010), partial least squares (Grigg and Grady, 2010), and detection of signal peaks (Liu and Duyn, 2013).

Early evidence of the neural basis of the two types of observed dynamic in resting state fMRI has suggested that different mechanisms may be behind them. First, using simultaneous recording of fMRI and local field potentials (LFP) from the rat brain, it was shown that changes in correlation between brain regions, measured in a sliding window over time, relate to changes in correlation in the electrical signal using the same window length (Thompson et al., 2013a): specifically band-limited power in the theta (4–8 Hz) and beta to gamma bands (25–100 Hz). This is similar to a relationship discovered between alpha (~10 Hz) band-limited power from electroencephalography (EEG) and resting state functional connectivity in humans (Chang et al., 2013). Second, using the same rat model as Thompson et al. and using electrodes and amplifiers that could record fluctuations down to the DC (0 Hz) component of the signal (Pan et al., 2013), correlations between infraslow LFP and fMRI matched the QPP observed in fMRI alone, and changes in strength of these patterns over time weakly but significantly correlated with the LFP signal directly (Thompson et al., 2013b). Preliminary evidence from an infraslow EEG study suggests that the correlation between the infraslow electrical signal and QPP in fMRI may exist in humans as well (Grooms et al., 2012).

Thus far, evidence has only linked QPP with infraslow fluctuations in neural electrophysiology (<1 Hz) and sliding window correlation with band-limited power of higher frequency fluctuations (>1 Hz), and not vice versa. Furthermore, the correlation pattern between band-limited LFP power and fMRI does not show a similar propagating wave as the infraslow potentials (Pan et al., 2011b). However, the relationship between these two scales of electrical activity (and how they relate to the resting state fMRI signal) is unknown and has not yet been systematically tested. Two possibilities exist; first, it is possible that sliding window correlation variations and QPP reflect the same underlying changes in the neural electrical signal, or second, the underlying processes that QPPs and sliding window correlations reflect could be independent and thus have independent relationships to dynamic resting state fMRI.

It has been hypothesized that the infraslow fluctuations observed in resting state fMRI originate in power fluctuation in higher frequency electrical activity: specifically, that the phase of the infraslow neural activity corresponds to specific power changes in a higher frequency band of neural activity, and the BOLD reflects the infraslow neural electrical band (Raichle, 2011). This is known as phase-amplitude coupling (Canolty and Knight, 2010). Most studies of phase-amplitude coupling have not examined infraslow phases; instead they have linked the phases of the equivalent of the EEG delta through beta bands (~1–25 Hz) to the amplitudes of the gamma band (25–100 Hz) in both non-invasive human EEG studies (Schack et al., 2002; Sederberg et al., 2003) and in invasive animal studies (Bragin et al., 1995; Chrobak and Buzsaki, 1998). However, some work has been done using infraslow EEG. Vanhatalo et al. observed coupling between the trough of the infraslow wave's phase and high amplitudes in higher frequency activity (Vanhatalo et al., 2004). Monto et al. found that high power in every high frequency band was coupled to the ascending part of the infraslow wave's phase (Monto et al., 2008). LFP and EEG are both aggregate and extracellular measures of neural activity, and thus LFP studies in animals can sometimes be used as homologs of EEG studies in humans, trading the direct applicability to humans for less noise and better spatial resolution (Buzsaki et al., 2012). The infraslow LFP signal also demonstrates a strong linear relationship with the local BOLD signal in matched frequencies (Pan et al., 2013; Thompson et al., 2013b). The infraslow phase-amplitude coupling seen in these EEG studies, when combined with the relationship between infraslow LFP and BOLD, thus suggests that a high frequency neural power vs. low frequency BOLD phase relationship may be possible.

The present study was done to test the hypothesis that previous results comparing resting state fMRI dynamics (such as QPP or changes in sliding window correlation) in the anesthetized rat in high frequencies (1–100 Hz) (Thompson et al., 2013a) and in infraslow frequencies (0.04–0.3 Hz) (Thompson et al., 2013b) may have had a common source due to phase-amplitude coupling. Infraslow (0–1 Hz) local field potential (LFP) phases and BOLD phases were compared to simultaneously recorded high-frequency LFP amplitudes (1–50 Hz). Interestingly, phase-amplitude coupling was only consistent under isoflurane anesthesia, not dexmedetomidine anesthesia. In a correlation analysis, under dexmedetomidine, there was no effect of either infraslow or high-frequency LFP on the other LFP band's correlation with BOLD. Under isoflurane, only the infraslow LFP had an effect on high-frequency LFP/BOLD correlations. These results indicate that the only phase-amplitude coupling that could be consistently observed was due to was due to neural suppression creating a burst state under isoflurane (Thompson et al., 2013b), suggesting that phase-amplitude coupling is not inherent to the resting state BOLD signal. This suggests that research into dynamic changes in resting state fMRI needs to consider multiple frequency bands in the underlying neural electrical activity, as different bands may contribute substantially different components to the observed functional networks in the BOLD. In particular, certain diseases or behaviors may be more strongly related to one scale of dynamics than another, explaining seemingly contradictory results in the resting state fMRI literature, which include greater activation in the default mode network being either helpful (Sadaghiani et al., 2009) or detrimental (Eichele et al., 2008) to a detection task and some diseases, such as Schizophrenia, showing both increases and decreases in functional connectivity (Garrity et al., 2007).

## Materials and methods

Except otherwise noted, all experiments were performed at Emory University in Atlanta, GA, and all data analysis was done using *MATLAB*.

### Infraslow LFP data collection (“bench” data)

All experiments were done in compliance with the Emory University Institutional Animal Care and Use Committee. Ten Sprague-Dawley rats (Male, Charles River Labs, ~350 g, ~70 days old) were anesthetized with 5% isoflurane anesthesia and were moved to a stereotaxic head-holder where their head was fixed centrally with ear bars. During surgery, isoflurane anesthesia was continued at 2–3.5%, at the minimum concentration that was needed to maintain a deeply anesthetized state with no reaction to toe-pinch. Temperature was monitored with a rectal temperature probe and heating pad feedback loop, and breath rate was manually timed.

Each rat's head was shaved and the skin opened in a rostral-caudal incision above the craniotomy area approximately 2 cm in length. Using a drill, two craniotomies were opened centered above the lower forelimb region of the primary somatosensory cortex (S1FL) in each hemisphere: 1 mm rostral of bregma and 4 mm lateral in each direction. Diameter of the craniotomy varied between 1 and 2 mm. Glass electrodes (1–5 MΩ impedance) were used, filled with 0.5 M saline solution and had a silver/silver chloride lead placed in them to facilitate electrical recording. Electrical recording was done using A-M systems model 3000 amplifiers lacking a highpass filter and capable of recording zero Hz signals. These amplifiers were used in direct current (DC) mode, to mitigate impedance dependent phase shift effects (Nelson et al., 2008). Data were visualized prior to recording at 5 kHz in *MATLAB* and were recorded also in *MATLAB* at 12 kHz. Electrodes were implanted in the center of the craniotomy, deviating by less than 0.5 mm to avoid a blood vessel if necessary. Electrodes were implanted at a depth of 1 mm to approximately level IV. This was verified by confirming that bursts seen under isoflurane featured deflections in the negative direction (Jones et al., 2004); if bursts did not deflect robustly in the negative direction, depth was varied up to ± 0.25 mm until robust negative deflection was observed.

For the first five rats, isoflurane was reduced to 2% and four 10 min LFP runs were recorded per rat in succession from bilateral S1FL. These rats were then euthanatized with an overdose of isoflurane by raising concentration to 5% and reducing air flow. Two 10 min LFP runs were then recorded from the dead rat as a control against inherent cross-frequency coupling in the signal. Breath rate was 73 ± 8 breaths per minute, temperature was 37.4 ± 0.6 °C (mean ± standard deviation).

For the second five rats, a bolus of 0.025 mg/kg dexmedetomidine was injected subcutaneously. After 10 min, isoflurane was discontinued and a continuous subcutaneous infusion of dexmedetomidine at 0.05 mg/kg/h was started. A minimum of 30 further minutes was waited until recording was begun, to reduce lingering effects of the isoflurane (Magnuson et al., 2014). Four 10 min LFP runs were recorded in succession from bilateral S1FL. Breath rate was 75 ± 11 breaths per minute, temperature was 37.2 ± 1.1°C (mean ± standard deviation). There was no significant difference in breath rate or temperature between anesthesia groups (*p* = 0.30 for breath rate, 0.22 for temperature, student's *t*-test, two tails, equal variance).

These data will be referred to as “bench” data.

### Simultaneous fMRI and infraslow LFP data from a previous study (“scanner” data)

Data from a previous study published in NeuroImage (Thompson et al., 2013b) by the authors of this study were also used. These included data recorded under isoflurane (4 rats, 17 runs) and dexmedetomidine (7 rats including one with data also recorded under isoflurane, 39 runs). Dexmedetomidine dosage was identical to data recorded for the present study, however a delay of at least 2 h after discontinuation of isoflurane was present in these data. Isoflurane concentration varied from 1.7 to 2.0%.

These data included LFP implanted in an identical location to the present study (interhemispheric S1FL) which was recorded simultaneously with a single fMRI slice from a 9.4 T MRI scanner (Bruker). fMRI were recorded using echo-planar imaging at *TR* = 0.5 s (2 Hz sampling rate), *TE* = 15 ms, 64 × 64 × 1 voxel matrix, 0.3 × 0.3 × 2 mm voxel size. This recorded the blood-oxygen level dependent (BOLD) signal. The single slice was positioned directly anterior to the location of the electrodes' tip and approximately in the coronal plane. For further details on simultaneous fMRI-LFP recording in rodents, see Pan et al. (2013), Thompson et al. (2013b) and the Journal of Visual Experiments video (Pan et al., 2010).

These data will be referred to as “scanner” data.

### Data pre-processing

LFP data were re-sampled to 1 kHz to reduce computation times. Each LFP data trace was visually inspected for presence of bursting (isoflurane) or sustained activity (dexmedetomidine), lack of amplifier saturation, lack of spikes due to head motion and (for scanner data only) successful removal of fMRI artifacts. These criteria resulted in removal of 1 entire run and removal of part of 3 additional runs from the bench data and removal of six entire runs from the scanner data.

BOLD data from simultaneous fMRI were cropped to include only the brain, motion-corrected and blurred with a Gaussian kernel with a full-width half-maximum of 0.5 mm (see Pan et al., 2013 for details). Each voxel had independently a linear de-trend performed to reduce drift artifacts, and was set to unit variance. On a per-run basis, a region of interest was drawn over S1FL in each hemisphere using a rat brain atlas as a guide (Paxinos and Watson, 2005). The mean signal was taken from this region of interest at each time point to produce a representative BOLD time series for S1FL in each hemisphere for each run. BOLD data were then resampled to 1 kHz to match LFP signals.

### Calculation of power spectra

To assess data consistency, power spectra were calculated for the time series from each LFP and each equivalent S1FL BOLD signal. Two spectra were created for each LFP signal, a low frequency spectrum from 0.01 to 1 Hz and a high frequency spectrum from 1 to 50 Hz. Only the 0.01–1 Hz spectrum was created for BOLD signals. Spectra were calculated using Welch spectra with 1000 frequency steps, 200 s long segments and 50% overlap. Mean power spectra were taken over all runs and electrodes for each combination of anesthesia, frequency band, and recording method.

### Phase-amplitude coupling

Phase-amplitude coupling, a form of cross-frequency coupling also known as nested oscillations, emerges when the phase of a particular oscillation in a signal corresponds to specific power changes (changes in maximum amplitude values) in a higher frequency in the same signal. A review paper by Canolty and Knight was consulted to determine the best method of cross-frequency coupling for use with the infraslow signal (Canolty and Knight, 2010). The phase-amplitude coupling method published by Tort et al. was chosen (Tort et al., 2010) because it is sensitive to the intensity of cross-frequency coupling, does not require visible peaks in power spectra, and can detect any form of phase-amplitude coupling where the histogram of high-frequency power vs. low-frequency phase is non-uniform; this includes high frequency power coupling at multiple phases of a single low frequency.

The algorithm from Tort et al. was used to calculate phase-amplitude coupling (Tort et al., 2010). Prior to running the algorithm, each signal was set to unit variance and zero mean. The original LFP signals were full band including all possible frequencies based on length and sampling rate. They were filtered twice to produce two new signals each: a low-frequency signal and a high-frequency signal. The Hilbert transformation (“Hilbert” function in *MATLAB*) was then calculated on each of the filtered signals to separate phase and amplitude. The amplitude was taken from the high-frequency signal and the phase from the low-frequency signal. Histograms were produced by taking the mean high-frequency amplitude value in several ranges of low-frequency phases. Twenty phase bins were used. Each histogram then had the Kullback-Leibler divergence (K-L) (Kullback and Leibler, 1951) calculated between it and a uniform distribution. The K-L values were converted to a modulation index between zero and one with zero indicating that the histogram matches a uniform distribution and one indicating it is as far from uniform as possible (a Dirac-like distribution). For details, see Figure 1 in Tort et al. (2010).

**Figure 1:**
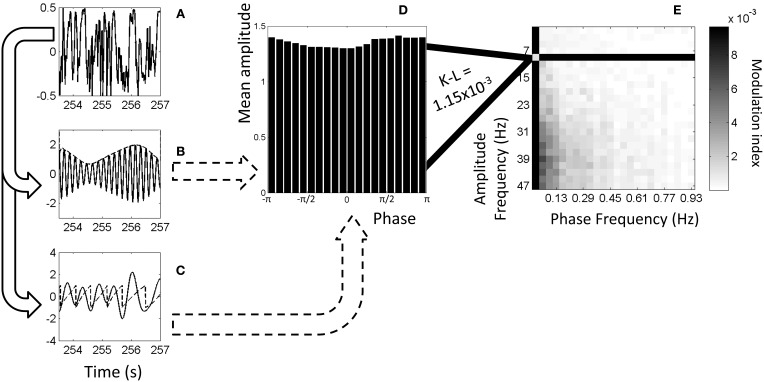
**Example of calculation of phase-amplitude coupling. (A)** Original, full-band electrophysiology signal recorded from left S1FL from a dexmedetomidine-anesthetized rat. **(B)** The signal filtered to 5–6 Hz (solid line) and the amplitudes from the Hilbert transformation (dashed line). **(C)** The signal filtered to 1–2 Hz (solid line) and the phases from the Hilbert transformation (dashed line). **(D)** Histogram showing mean amplitude corresponding to each phase bin. Each point on the dashed line from **(B)** is put into a bin based on the equivalent phase on line **(C)** and averaged with other points in that bin. (For illustration purposes only, this histogram and later is from 9–11 Hz amplitude frequency and 0.01–0.05 Hz phase frequency instead of exactly the dashed lines' frequencies). **(E)** Comodulogram showing modulation index at each range of phase frequencies and amplitude frequencies, darker colors indicating greater phase-amplitude coupling. Each point on this map is color-coded representing a modulation index calculated from K-L distance on a histogram such as the one shown in **(D)**. All comodulograms for a given condition are combined and tested at each point against a similar distribution of shuffled data to find statistical significance.

The range of low frequencies used for phases was from 0.01 to 0.97 Hz in steps of widths of 0.04 Hz (e.g., 0.01–0.05 Hz, 0.05–0.09 Hz, etc.). The range of high frequencies used for amplitudes was from 1 to 49 Hz in steps of widths of 2 Hz (e.g., 1–3 Hz, 3–5 Hz, etc.). In every case, the filter used was a boxcar wave (a Fourier-domain multiplicand) because this type of filter could be processed quickly in *MATLAB*. (Tests with other standard filters were several orders of magnitude slower) The phase cycle was defined in radians from -π to π, as per *MATLAB* standards.

Normalized K-L values resulting from each phase frequency/amplitude frequency combination were plotted in 2D maps known as comodulograms (Canolty et al., 2006). In these maps, the color scale from dark (high amount of coupling) to light (low amount of coupling) represents the normalized K-L value, the horizontal axis represents the frequency of the phase signal and the vertical axis represents the frequency of the amplitude signal.

In addition to standard use of the Tort et al. algorithm (Tort et al., 2010), another test was run on scanner data. The same calculations were done for the high-frequency signal, but the BOLD signal from S1FL in the same location as the LFP was filtered to the same frequency band as the LFP would have been, and substituted for it in the low-frequency signal for calculation of phase. After this step, the histograms and comodulograms were created as normal. Thus, this method showed modulation between the fMRI signal's low-frequency phase and the LFP signal's high-frequency amplitude. This was done both with no shift between LFP and BOLD, and with a 4s shift (isoflurane) or 2.5s shift (dexmedetomidine) forward in time for BOLD data to approximate the hemodynamic response function seen in previous work (Pan et al., 2011b).

Figure 1 illustrates the algorithm used to calculate phase-amplitude coupling and the creation of a comodulogram.

### Significance testing

Comodulograms were tested for significance by comparison to surrogate data. The surrogate data were generated identically to the actual data, except taking the low-frequency signal from the next scan in succession, circularly (e.g., scan one's amplitude goes with scan two's phase, etc.). This preserved basic signal characteristics but removed any time-locked information between the two signals, including phase-amplitude coupling. This removal occurs because the phase-amplitude coupling is calculated on an instant by instant basis and, as can be seen in Figure 1A, any given frequency's phase will vary throughout the signal. As each histogram is calculated at every point across the signal, one mismatch is sufficient to eliminate effects of sinusoids transiently aligning to each other.

Comodulograms, calculated on a per-scan, per-electrode basis, were divided into sets based on how they were recorded (bench LFP, scanner LFP, or scanner LFP vs. scanner fMRI) and the condition (dexmedetomidine, isoflurane or a dead rat). Prior to using a statistical test, the logarithm was taken of K-L values to transform them to a normal-like distribution (see Data sheet 1). Two types of statistical test were calculated between the actual and surrogate data distributions for each frequency location. First, a student's *t*-test with two tails and equal variance, and second, a two-sided Kolmogorov-Smirnov (*KS*) test.

Probability (*p*) values for all frequency locations and all sets of comodulograms were combined and corrected for multiple comparisons using sequential goodness of fit (SGoF) at 0.05 (Carvajal-Rodriguez et al., 2009). SGoF is a binomial method which searches for a sufficiently large cluster of small *p*-values, rather than sufficiently small individual *p*-values. Therefore, it corrects against Type I error (false positive) without greatly increasing Type II error (false negative). Two statistical families were used: all LFP phase vs. LFP amplitude data was the first family, and all fMRI phase vs. LFP amplitude was the second family.

### fMRI-LFP correlation

To test the relationship between the low frequency and the high frequency components of the LFP signal in the context of fMRI, correlation between LFP and the BOLD signal from S1FL was calculated. The filters were taken from the steps used to calculate comodulograms, except 2 additional steps in each direction total were taken (equivalent to a 5 × 5 square on a comodulogram) to increase the signal present. The low frequencies were selected as 0.01–0.17 Hz for isoflurane and 0.09–0.29 Hz for dexmedetomidine, the high frequencies were selected as 15–25 Hz to match beta power for isoflurane and 1–9 Hz to match delta through theta power for dexmedetomidine. These frequency ranges were chosen so as to contain the frequencies of peak coherence observed in previous work (Figures 3, 4 from Pan et al., 2013). For each run and electrode separately, the LFP and equivalent BOLD signals were filtered to the low frequency band with a hard-edged Fourier filter to produce low-frequency BOLD and low-frequency LFP signals. Also for each run and electrode, the LFP was filtered to the high frequency band and the absolute value of the Hilbert transformation was taken to produce the high frequency power LFP signal. All three signals from each run (low-frequency LFP, low-frequency BOLD, and high-frequency power LFP) were set to zero mean and unit variance.

Two correlation metrics were calculated for each run and electrode. First, standard correlation was calculated between the low-frequency LFP and the low-frequency BOLD and between the high frequency LFP power and the low-frequency BOLD. Second, a partial correlation analysis was done. Like standard correlation, partial correlation is a measure of the amount of similarity between two time series, but in partial correlation a third time series is specified as a “controlling variable,” and the effects of it are removed from the other two time series for the purposes of correlation. Here, partial correlation was calculated between the low-frequency LFP and the low-frequency BOLD, with the high-frequency LFP power acting as a controlling variable, and between the high frequency LFP power and the low-frequency BOLD with the low-frequency LFP acting as a controlling variable. Every correlation was done at time shifts from the BOLD prior to the LFP by 5 s (negative shifts) to the BOLD after the LFP by 10 s (positive shifts, the direction of the canonical “hemodynamic response”). To reduce computation time and the number of tests overall, only every 250th shift (4 shifts/s, 60 shifts total) were tested. LFP signals were shifted together, i.e., the infraslow and high frequency LFP were shifted relative to the BOLD but not relative to each other. All correlation values were converted to *z*-scores that would approximate a N(0,1) distribution if no correlation existed, using equation 1 from Thompson et al. (2013a). For both standard and partial correlation, separate analyses were performed identically using either Pearson's or Spearman's correlation coefficients.

Two types of statistical significance were tested. First, for each condition (two anesthesias, high frequency, and low frequency) and for every time shift data from different electrodes and runs were pooled and a student's *t*-test (two-sample, two tails and equal variance) was performed between the *z*-scores from standard correlation and the *z*-scores from partial correlation. The *p*-values resulting from these *t*-tests were pooled and controlled for multiple comparisons using SGoF at 0.05, considering each frequency band and anesthesia combination to be a statistical family. Second, the calculation of *z*-scores was repeated with shifted data (see previous section for shifting process) to create surrogate data. For each condition (two anesthesias, high frequency, and low frequency), for every time shift and for partial vs. full correlation data from different electrodes and runs were pooled and a student's *t*-test (two-sample, two tails, and equal variance) was performed between the *z*-scores from actual data and the *z*-scores from surrogate data. *P*-values resulting from these *t*-tests were pooled and controlled for multiple comparisons using SGoF at 0.05, considering each frequency band and anesthesia combination to be a statistical family.

## Results

### Power spectra

**Figure 2:**
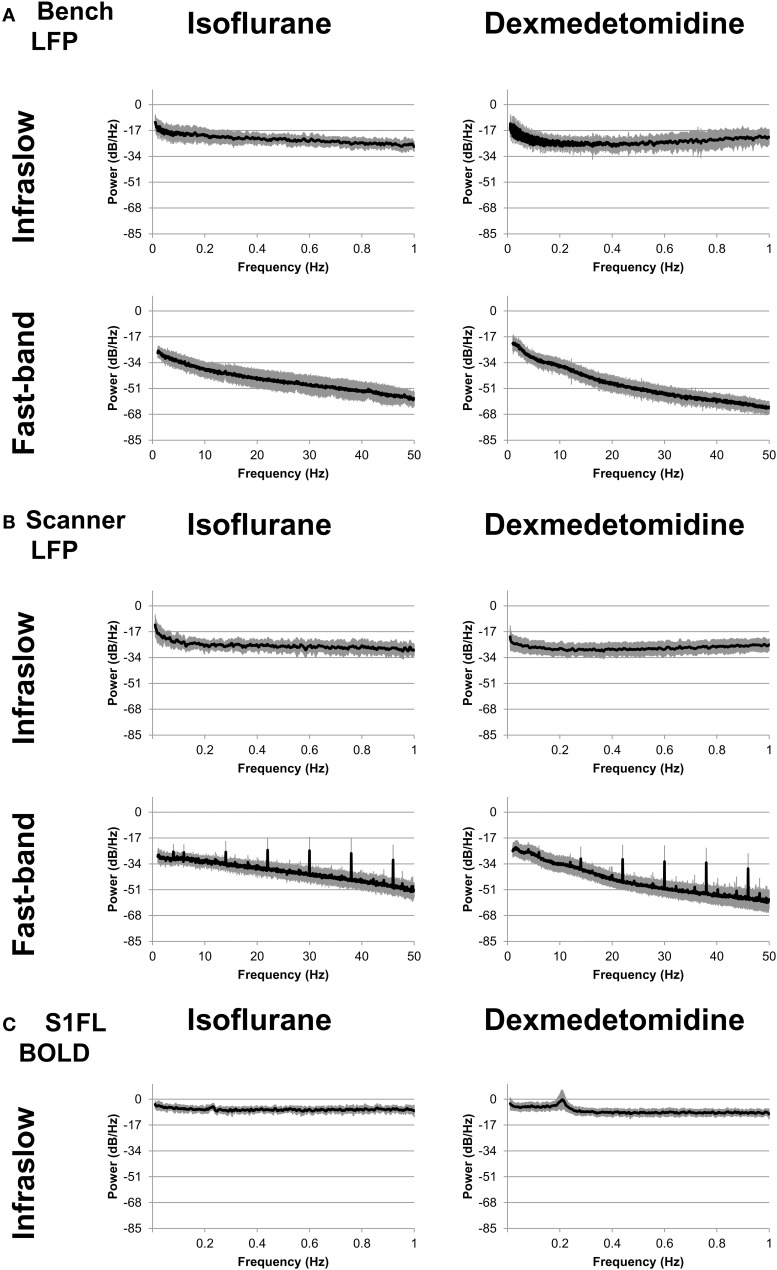
**Power spectra**. Spectra were calculated separately for fast-band (1–50 Hz, bottom row) and infraslow (0.01–1 Hz, top row) frequencies, and anesthesia of either isoflurane (left column) or dexmedetomidine (right column). **(A)** “Bench” LFP data recorded outside the fMRI scanner. **(B)** “Scanner” LFP data recorded while fMRI scanning was present. While the LFP recorded during BOLD imaging shows noisier spectra, in general spectra are similar across recording methods for each anesthesia. **(C)** fMRI data recorded during simultaneous LFP recording.

Power spectra were generated for each electrode and run and averaged across each combination of modality/recording method (fMRI from the simultaneous experiment, LFP from the simultaneous experiment, and LFP from the bench experiment), anesthesia (isoflurane and dexmedetomidine), and two bands (0.01–1 Hz and 1–50 Hz). Average results are shown in Figure 2. For the low frequency band, each condition shows relatively constant power with an average profile that appears similar for both bench and scanner LFP under isoflurane (a gradual decrease) and dexmedetomidine (A gradual decrease until approximately 0.2 Hz, then a gradual increase). fMRI power is largely consistent across the low frequency band, with the exception of a peak at approximately 0.2 Hz which is the subject of another study by the authors of this study in both cerebral blood volume (Magnuson et al., 2010) and BOLD imaging (Pan et al., 2011a; Magnuson et al., 2014). Power spectra in the high frequency LFP are generally consistent for a given anesthesia type across recording methods, however the in-scanner recording shows a large spike at ~6 Hz and then every ~8 Hz thereafter. Unlike low-frequency power, high-frequency power shows a steady decrease in power as frequency decreases. This may indicate a 1/f or “pink noise” characteristic, typical of neural signals.

The spikes seen in the in-scanner recorded LFP data are due to the artifact produced in the LFP signal when the gradient coils switch. The magnetic induced artifacts in raw recordings during fMRI scanning were minimized after noise removal; the process is detailed in Pan et al. (2010, 2011b, 2013). However, this process is not perfect and occasional periodic noise can remain in some scans, resulting in this artifact. This factor was a major reason for including the bench data in this study, as it completely lacks this artifact. As results in the following sections are generally similar for both bench and scanner data, it is unlikely this artifact influenced the results.

Overall, power spectra results show that bench and scanner LFP recording have a similar profile, more consistent power in the low frequency (0.01–1 Hz) band and a steady decrease in power as frequency increases in the high frequency (1–50 Hz) band.

### Phase-amplitude coupling

**Figure 3:**
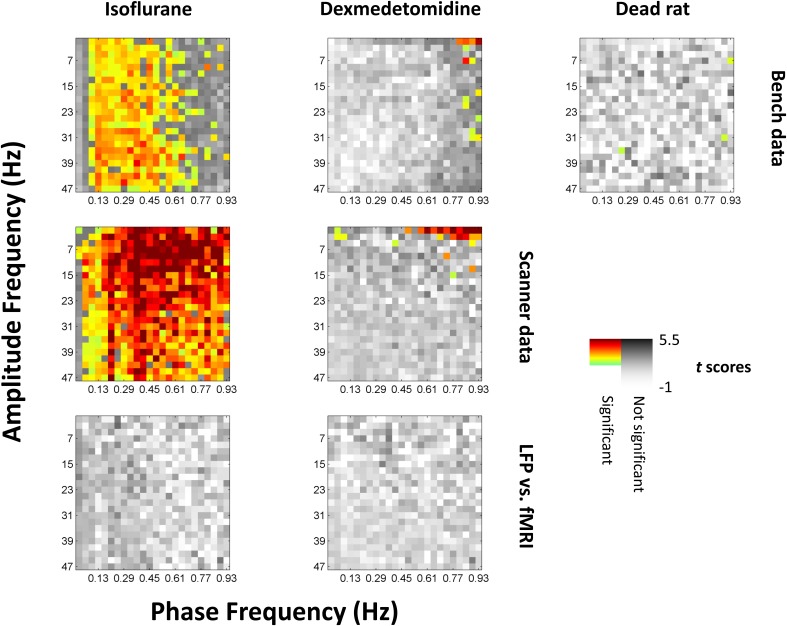
**Significance of phase-amplitude coupling as tested against shuffled data**. All values from comodulograms were compared vs. data where the phase signal had been replaced with the phase signal from the next scan under the same conditions. Grayscale colors are *t* scores with darker being a greater chance of rejecting the null hypothesis. Colors indicate significance and a greater average value (more coupling) than shuffled data, with light green indicating low *t* scores to dark red indicating high *t* scores. The first row represents data recorded outside the fMRI scanner, the second row represents data recorded inside the scanner during simultaneous imaging. The third (bottom) row is the same as the second row but has had the phase signal replaced with the equivalent fMRI signal shifted by 4s (isoflurane) or 2.5s (dexmedetomidine) to approximate the hemodynamic delay; there were no significant results for this row, and the same case but without a hemodynamic shift. Left column is isoflurane anesthesia (*N* = 21 for bench, 17 for scanner, times 2 electrodes), middle column is dexmedetomidine anesthesia (*N* = 20 for bench, 39 for scanner, times 2 electrodes), right column is following euthanatization of the rat (*N* = 10 times 2 electrodes, only available outside the fMRI scanner). Isoflurane shows broad significance outside the lowest infraslow frequencies, while dexmedetomidine shows significance only at high infraslow frequencies and low fast-band frequencies. The original comodulograms are shown in Data sheet 2 (actual data) and Data sheet 3 (shuffled data).

Comodulograms were generated for each electrode and with phase frequencies from 0.01 to 0.97 Hz in steps of widths of 0.04 Hz and amplitude frequencies from 1 to 49 Hz in steps of widths of 2 Hz (e.g., 1–3 Hz, 3–5 Hz, etc.). Comodulograms were generated for each electrode and run and averaged across each combination of recording method (fMRI, scanner LFP that was simultaneous with fMRI, and bench LFP that was not) and condition (isoflurane and dexmedetomidine, and dead rat). Comodulograms generated from actual data are shown in Data sheet 2. Comodulograms generated from surrogate data where phase and amplitude signals were mismatched are shown in Data sheet 3. Student's *t*-tests were performed between these distributions at each phase vs. amplitude point and the *t* scores resulting are shown in Figure 3.

Statistical significance was found by correcting for multiple comparisons using SGoF at 0.05 on *p*-values resulting from the *t*-tests, resulting in a cutoff of *p* ≤ 0.011. Significant squares are shown on Figure 3 in color with warm colors indicating higher *t*-values. 904 points were noted as significant including 330 for bench-recorded LFP under isoflurane, 532 for scanner-recorded LFP under isoflurane, 14 for bench-recorded LFP under dexmedetomidine, 25 for scanner-recorded LFP under dexmedetomidine, and 3 for the dead rat. With the exception of the dead rat, where the 3 points are scattered, the significant results tend to be clustered. Under isoflurane they are in a vertical band encompassing all high frequencies that either only excludes the lowest frequencies (for scanner recorded LFP) or excludes both the upper and lower end of the 0.01–1 Hz band (for bench recorded LFP). Under dexmedetomidine, the significant results are clustered at either the lowest high frequencies (for scanner recorded LFP) or the highest low frequencies (both scanner and bench recorded LFP).

Significance found from *KS*-tests was in the same areas as were found for *t*-tests. The results are shown in Data sheet 4.

When the fMRI signal was used as a surrogate for the phase's signal, either with or without a 4s (isoflurane) or 2.5s (dexmedetomidine) shift for BOLD, it did not show any statistically significant phase coupling to amplitudes from the LFP signal.

### Consistency of phase-amplitude coupling

The K-L scores shown on comodulograms (Data sheets 2, 3) and thus the statistical differences between them (Figure 3) only reflect the magnitude of phase-amplitude coupling, but not the type. Thus, Figure 3 shows which frequency pairs produce histograms that, on average, differ from uniform more than expected (i.e., vs. the shuffled data). However, Figure 3 does not give any indication that these deviations from uniform are consistent on a trial by trial basis, i.e., consistent in which parts of the phase couple to which amplitudes. A physiological source for phase-amplitude coupling would be expected to show consistent coupling between specific infraslow frequencies' phases and specific higher frequency amplitudes. However, spurious phase-amplitude coupling resulting from differences in the recording of the signals themselves (e.g., differences in signal to noise ratios scan-to-scan) or resulting from lack of statistical power due to methodological limitations (see Section Technical Limitations) may have resulted in statistically significant K-L scores on average, but with no consistent relationship between which phases correspond to higher amplitudes. Therefore, further investigation was warranted to see if the statistically significant phase-amplitude coupling actually reflected a consistent relationship.

**Figure 4:**
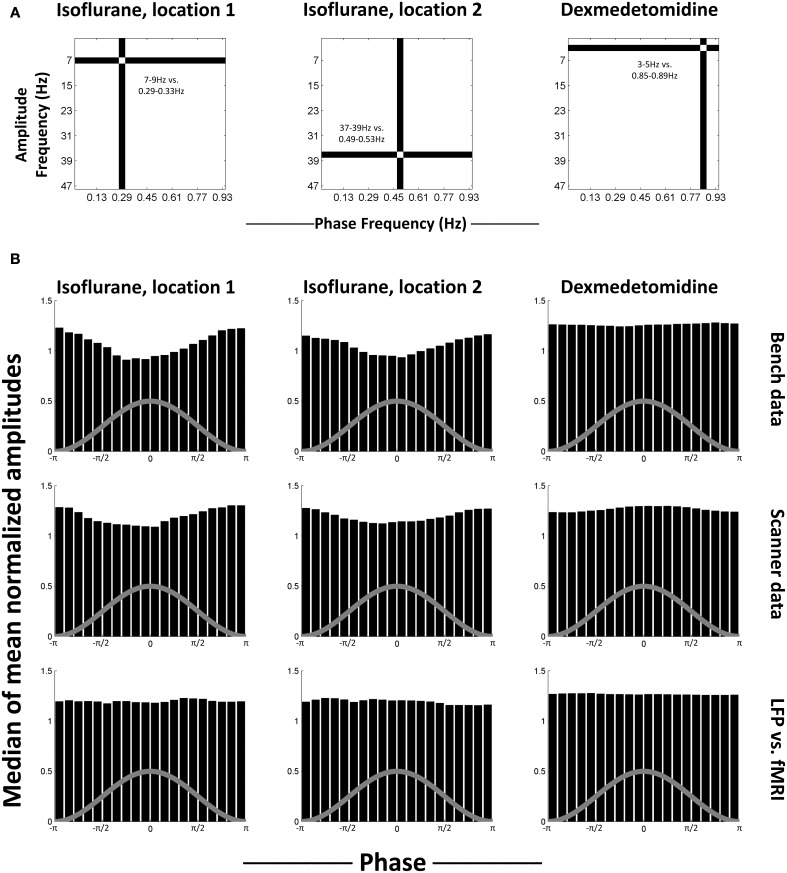
**Median histograms. (A)** Location of the phase frequency and amplitude frequency selected as representative and used to calculate the histograms shown in each column. These locations on the equivalent comodulograms from Figure 3 are shown. **(B)** Median histograms for selected, statistically significant locations in comodulograms. Histograms shown are the median of all histograms for that condition. Each bin in the original histograms contained the mean of normalized amplitudes (ordinate) for the fast frequencies that matched that bin's phases (abscissa) for the slow frequencies. Each histogram represents one point on a comodulogram, being converted to a modulation index by the K-L distance from a uniform distribution. The rows are, in order, data recorded outside the fMRI scanner, data recorded during simultaneous image acquisition and the last row is the same as the middle row, except with phase data replaced with equivalent simultaneously recorded fMRI data. The first two columns are locations that were significant in isoflurane comodulograms vs. shifted data (*N* = 21 for bench, 17 for scanner, times 2 electrodes), the third column is a location that was significant in dexmedetomidine comodulograms vs. shifted data (*N* = 20 for bench, 39 for scanner, times 2 electrodes). The gray line on each histogram is a descriptive picture of the phase cycle.

To test for a consistent relationship in phase-amplitude coupling, select comodulogram locations (phase-amplitude pairs) were examined as representative. The locations that were selected for each anesthesia were statistically significant for both bench and scanner LFP recording. This is because the use of K-L values ensures that the furthest from uniform histograms will be marked as statistically significant. Therefore, non-statistically significant histograms will be much closer to uniform and thus less interesting. The selected locations were 0.29–0.33 Hz phases vs. 7–9 Hz amplitudes and 0.49–0.53 Hz phases vs. 37–39 Hz amplitudes for isoflurane and 0.85–0.89 Hz phases vs. 3–5 Hz amplitudes for dexmedetomidine (Figure 4A). For each location, the histograms of mean normalized amplitude vs. phase were calculated.

Figure 4B shows the median of generated histograms for each location (two isoflurane locations, one dexmedetomidine location) and each recording method (bench LFP, scanner LFP, scanner LFP vs. simultaneous fMRI). Median (50th percentile) was used to find average values across histograms without assuming a distribution. The median histograms for the LFP signal under isoflurane anesthesia show a clear phase-amplitude relationship with the peak of the phase in the slow frequency corresponding to a decrease in mean amplitude at the high point in the cycle for phase. This is likely a combination of two factors. First, isoflurane induces a “burst state,” in other words, alternation between periods of suppression and periods of activity (see Data sheet 5). Second, as bursting was in the negative direction, minimum high frequency power corresponds to the peak of the infraslow phase (Pan et al., 2013). The median histograms for the LFP signal under dexmedetomidine appear like a uniform distribution. The histograms generated when the fMRI signal was used to replace the low-frequency LFP used to calculate the phase are also uniform, and under both anesthesia. The uniformity of these histograms indicates that, while some individual trials may differ from surrogate date, no consistent trend toward consistent phase-amplitude coupling across trials.

**Figure 5:**
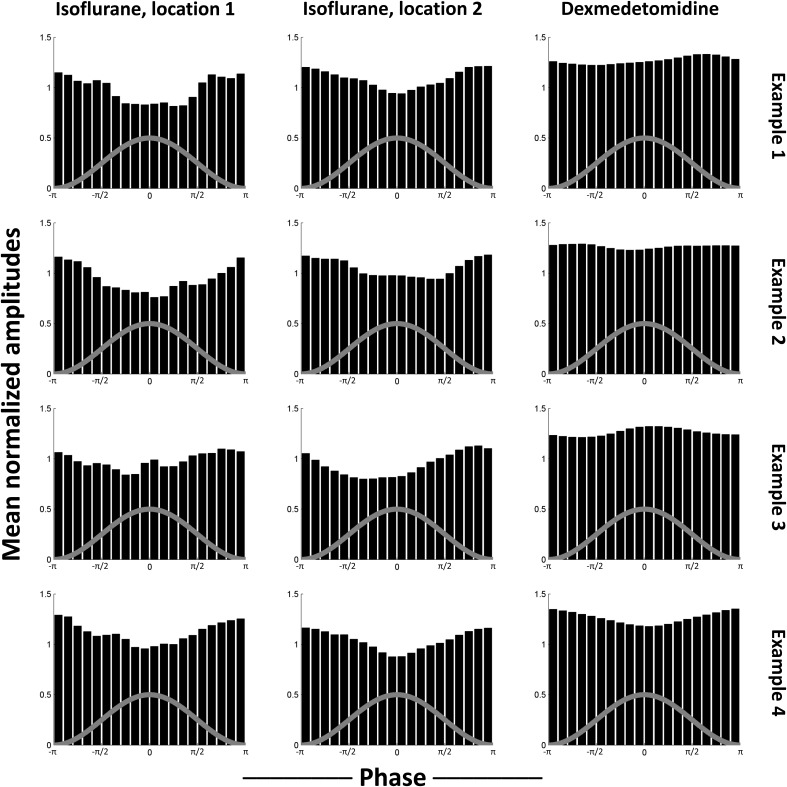
**Examples of individual scans' histograms**. Four individual scans were selected as representative from each case tested in Figure 4. The first two columns are locations that were significant in isoflurane comodulograms vs. shifted data (*N* = 21 for bench, 17 for scanner, times 2 electrodes), the third column is a location that was significant in dexmedetomidine comodulograms vs. shifted data (*N* = 20 for bench, 39 for scanner, times 2 electrodes). The comodulogram locations are identical to those in Figure 4 from the same columns. Each row is one representative example scan from bench data, using the electrode implanted into left S1FL. Note that, while noisy, histograms resulting from data recorded under isoflurane all are generally similar to the median histogram shown in Figure 4, with a trough at approximately the same phase, while histograms resulting from data recorded under dexmedetomidine show peaks at different phases, which is why slight significance was observed despite the overall median being uniform (Figure 4). The gray line on each histogram is a descriptive picture of the phase cycle.

As the median results shown in Figure 4 could obscure individual variation, phase-amplitude histograms from individual runs are shown in Figure 5. Four runs' results are shown from bench-recorded LFP at the same two locations in the comodulogram under isoflurane and the single location under dexmedetomidine. While individual histograms vary, all isoflurane histograms clearly show the trough in the mean high-frequency amplitude around the peak of the waveform for low-frequency phase. Dexmedetomidine, however, is inconsistent, showing sometimes a similar histogram to what was seen under isoflurane, sometimes the inversion of it, and sometimes a completely different histogram. This explains why the median histograms shown for dexmedetomidine in Figure 4 were near-uniform: the profile of phase-amplitude coupling under isoflurane is consistent, under dexmedetomidine it is not.

Individual histograms from the non-significant case where amplitudes from high frequency LFP were compared to infraslow phases from BOLD are shown in Data sheet 6. Here, no trend can be observed whatsoever. This suggests that the lack of statistical significance in this case indicated a lack of interesting phase-amplitude coupling, rather than sub-threshold but consistent results.

### fMRI-LFP correlation

Standard (Pearson or Spearman) correlation was calculated between the LFP signal's low frequencies (0.01–0.17 Hz for isoflurane, 0.09–0.29 Hz for dexmedetomidine) or the LFP signal's high frequency powers (15–25 Hz for isoflurane, 1–9 Hz for dexmedetomidine) and the fMRI BOLD signal local to the electrode (S1FL BOLD). Frequency ranges were chosen to include previously observed significant coherence (Pan et al., 2013). Partial correlation was also calculated. Like standard correlation, partial correlation is a measure of the amount of similarity between two time series, but in partial correlation a third time series is specified as a “controlling variable,” and the effects of it are removed from the other two time series for the purposes of correlation. Thus, if a partial correlation value is lower than the equivalent standard correlation, it suggests that part of this correlation may have been due to shared variance common to the controlling variable. If such a reduction is statistically significant it indicates that, while the two signals are still correlated, this relationship is not independent of the third signal. Conversely, no change, or an increase in the correlation coefficient under partial correlation suggests linear (and/or rank) independence, and thus a relationship between the first two time series that is possibly independent of the controlling variable. A significant reduction observed in partial correlation as compared to regular correlation indicates that the controlling variable modulates the correlation between the correlated variables.

**Figure 6:**
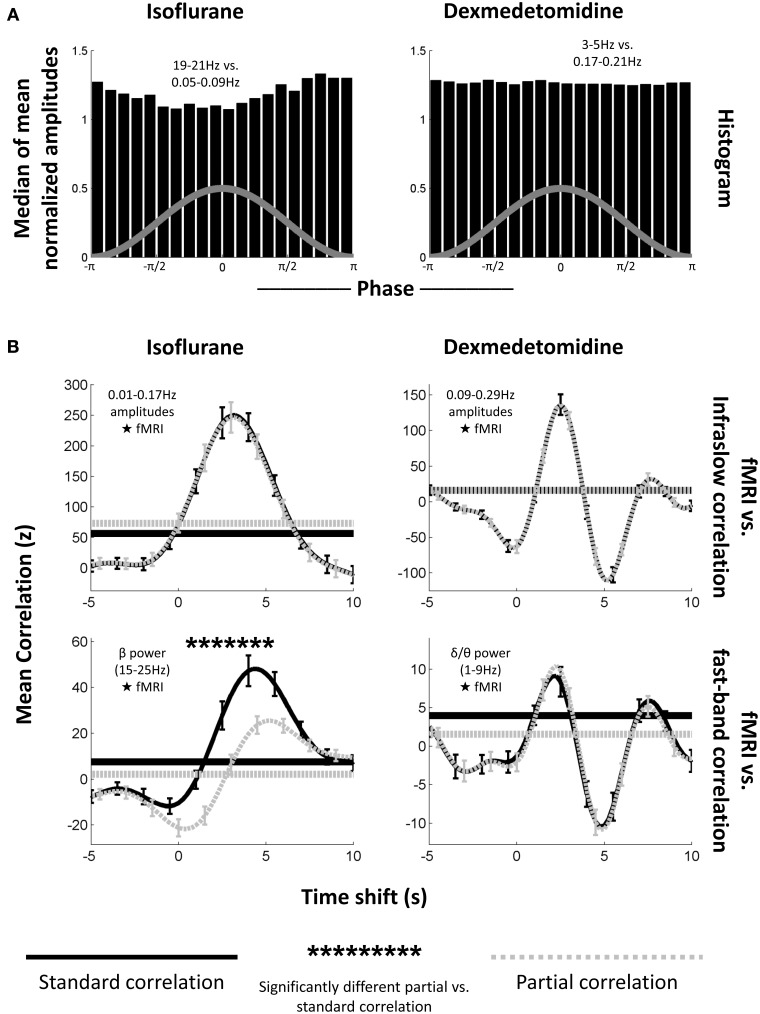
**Phase-amplitude coupling's effect on LFP-fMRI correlations. (A)** Histograms, as from Figure 4, showing median phase-amplitude coupling at the low frequency vs. high frequency location for isoflurane (left) and dexmedetomidine (right). Locations in the infraslow and EEG-band frequencies in the LFP were chosen because of their previously demonstrated correlation to the fMRI signal (Pan et al., 2013). Isoflurane is deviating from a uniform distribution as seen previously (Figure 4), but dexmedetomidine is not. **(B)** Plots of correlation (ordinate) between the LFP and fMRI signals at multiple time shifts for the fMRI signal (abscissa) with positive shifts indicating that fMRI has been shifted backward in time so that if fMRI was following the LFP, they would be aligned. Solid black lines are standard correlation between the electrophysiological signal and the fMRI, dashed gray lines are partial correlation between the same signals where the other electrophysiological signal has been included as a controlling variable (e.g., when the correlation with the low-frequency signal is plotted, the high frequency signal has been used as a controlling variable). Horizontal lines indicate the significance cutoff: values above the horizontal lines of the same style are significant (only positive correlations were significant). Error bars indicate one standard error of the mean. Asterisks above the plots indicate a significant difference between partial correlation values and regular correlation values at that time shift. The first column is under isoflurane anesthesia (*N* = 17 times 2 electrodes), the second column is under dexmedetomidine anesthesia (*N* = 39 times 2 electrodes). The first row is mean correlation between fMRI and amplitudes of matched infraslow frequencies, the second row is mean correlation between fMRI, and power in fast-band frequencies. Note that the only significant difference between regular and partial correlation occurs for fast-band frequencies under isoflurane. This supports the results of the significance testing of the comodulograms (Figure 3), and suggests that phase-amplitude coupling only plays a large role in fMRI-LFP correlations under isoflurane when EEG-band frequencies are tested.

Here, partial correlation was calculated identically to regular correlation, except with the LFP signal's high frequency powers acting as a controlling variable for the infraslow LFP signal's correlation with BOLD, and vice-versa. When correlating with the infraslow signal directly, correlation coefficients were inverted for plotting to follow the convention of Pan et al. (2013). Pan et al. did this due to the electrodes being implanted in approximately layer IV of the cortex and bursting being in the negative direction (Jones et al., 2004). These pairings of high vs. low frequencies were selected based on high LFP-fMRI coherence observed in previous work (Pan et al., 2013) and also showed similar phase-amplitude coupling to what was seen in the previous section (Figure 6A).

Figure 6B shows the average result for Pearson correlation coefficients across all runs and electrodes, separated by frequency band (low frequency amplitude vs. BOLD and high frequency power vs. BOLD) and anesthesia (isoflurane and dexmedetomidine). Correlation is plotted vs. the amount forward in time the LFP signal was shifted. In every case, both partial and standard correlation had a statistically significant peak at a positive shift (BOLD events occurring after LFP events) when tested against surrogate data (0.015 ≤ *p* ≤ 5.5 × 10^−4^ for each case). This result suggested a hemodynamic response to neural activity (Magri et al., 2012). Under dexmedetomidine, a smaller significant peak was also seen following the main peak. Peak locations were 3.25 s with a rise time of ~5.75 s for low-frequency electrophysiology under isoflurane, 4.5 s (regular correlation) or 5.25 s (partial correlation) with a rise time of ~5 s for high-frequency electrophysiology under isoflurane, a large peak at 2.5 s with a rise time of ~2.75 s followed by a smaller peak at 7.75 s for low frequency electrophysiology under dexmedetomidine, and a large peak at 2.25 s with a rise time of ~3 s followed by a smaller peak at 7.5 s. These peaks are at approximately the same time shifts as have been reported previously in studies of the LFP-fMRI relationship in rodents; see Pan et al. (2011b) for a discussion of the difference between anesthetics (Pan et al., 2011b, 2013). All significant values were positive, no negative correlations were significant despite a two-tailed *t*-test having been used.

Results using Spearman correlation coefficients were very similar to Pearson correlation coefficients and always reported similar time shifts for statistical significance. These results are shown in Data sheet 7.

Distributions of *z*-scores from different runs and electrodes, within each anesthesia, frequency band and time shift, were tested to see if a difference existed between regular and partial correlation. The only statistically significant difference was seen for correlation between BOLD and LFP high frequency power with LFP low frequencies acting as a controlling variable under isoflurane (Pearson correlation coefficients, 19 significant time shifts, *p* ≤ 3.7 × 10^−3^, Spearman correlation coefficients, 18 significant time shifts, *p* ≤ 8.52 × 10^−3^). This is likely due to the burst state where periods of activity and inactivity would affect both high and low frequency neural activity (Data sheet 5), and therefore create shared variance. This difference lowered the peak height of LFP-fMRI correlation in the partial correlation plot vs. time shift, however partial correlation was still significant even after the peak was lowered. This suggests that, despite shared variance between frequencies due to the bursting, some independence in hemodynamic vs. electrophysiological correlations existed between the different bands.

## Discussion

### Summary

Previous studies have shown a relationship between the spontaneous fMRI signal and both the infraslow (<1 Hz) and band-limited power of faster (1–100 Hz) electrical activity. To investigate if these relationships may be coming from a common source, phase-amplitude coupling was calculated from both data recorded outside the fMRI scanner (“bench” data) and during simultaneous fMRI (“scanner” data) from the primary somatosensory cortex of rats under two different anesthetic agents (isoflurane and dexmedetomidine). The phase components were taken from the 0–1 Hz infraslow component of the electrical signals, and the amplitude components were taken from the 1–50 Hz component. Results were visualized as comodulograms with “phase frequencies” (the frequencies used to calculate phases) as the horizontal axis and “amplitude frequencies” (the frequencies used to calculate amplitudes) as the vertical axis. Significance was tested between actual data and data with mismatched sources for phase and amplitude.

The results indicated that phase-amplitude coupling existed under isoflurane only due to the general burst-suppression of that anesthesia, and did not consistently exist under dexmedetomidine (Figure 3).

Under isoflurane anesthesia, significant phase-amplitude coupling was seen broadly at all except the lowest amplitude frequencies. Two representative phase frequency/amplitude frequency pairs were examined in detail by producing the specific histograms of median amplitudes (normalized average values) vs. specific phase values (from -π to π). Under isoflurane, the observed phase-amplitude coupling was highly consistent, showing a trough around the phase of the low frequency's signal peaks. This inverted relationship (low amplitudes in the infraslow frequencies associated with high power in the higher frequencies) is likely due to the “burst state” induced by isoflurane; periods of neural suppression followed by periods of high activity, with bursts in the negative direction. Example LFP traces are shown in Data sheet 5 which clearly illustrates this differences between anesthetics. As negative bursts were ensured by this study's protocol (see Methods), and as all neural frequencies were thus periodically suppressed and bursting, this is likely the explanation for the large, unspecified bands of phase-amplitude coupling observed. This follows from results seen in a previous simultaneous fMRI-LFP rat study under isoflurane (Pan et al., 2011b) where all frequencies were shown to be coupled to BOLD.

Under dexmedetomidine, significant phase-amplitude coupling was only seen at the highest phase frequencies and lowest amplitude frequencies. One specific phase frequency/amplitude frequency was examined in detail by producing the specific histograms of median amplitudes (normalized average values) vs. specific phase values (from -π to π). Under dexmedetomidine, results varied from scan to scan, and had no consistent trend (Figures 4, 5). Dexmedetomidine has a very different mechanism than isoflurane (see Appendix A of Thompson et al., 2013b) and does not induce a burst state. This may be why no consistent phase-amplitude coupling was seen.

Spurious phase-amplitude coupling was seen at only three scattered points in the data recorded from a dead rat (Figure 3, far right column). These points are not significant on their own, but, as the dead rat's data were clustered with living rats for significance testing, a few points fell into the significant cluster with data from living rats. These points are widely separated and indicate random noise rather than any actual systemic bias.

A correlation analysis between fMRI, fast LFP bands, and slow LFP bands was also performed (Figure 6). All correlation tests showed a statistically significant peak at a time shift approximating the hemodynamic response for both frequency bands and both anesthesia. The time shifts where LFP correlates with BOLD are close to those observed in simultaneous experiments of non-human primates (Shmuel and Leopold, 2008; Magri et al., 2012). When the effect of the high frequency LFP on the infraslow LFP's correlation with fMRI was considered (using partial correlation), or vice versa, an effect was shown only under isoflurane, and only in the direction of the infraslow LFP influencing the high frequency LFP's correlations. However, even when accounting for the effect of infraslow LFP on the correlation between beta power in LFP and BOLD, there remained a (smaller) peak of significant correlation (Figure 6/Data sheet 7, bottom left, dashed line). This result suggests that, while the periodic neural suppression of the burst state under isoflurane may induce some LFP-fMRI correlation, it alone does not account entirely for the LFP-fMRI correlation seen under isoflurane. Under dexmedetomidine, it is not a factor and the relationship between infraslow LFP and fMRI appears to be independent of that between higher frequency LFP power and fMRI.

In addition to comparing different bands of the electrical signal, the high frequency components of the electrical signal were compared to low-frequency components of a simultaneously recorded fMRI signal from the same brain area. However, no significant results were seen when this was done (Figure 3, bottom row), or even a trend toward significance (Data sheet 6). This is in contrast to existing hypotheses about the origin of the resting state fMRI signal (see section Previous Studies of Infraslow Phase vs. High Frequency Amplitude Coupling). The lack of phase-amplitude coupling between fMRI and LFP (Figure 3, bottom row) vs. the presence of significant fMRI-LFP correlations (Figure 6) suggests that rather than only the phase of the infraslow fMRI signal being related to electrical potentials, the amplitude of the infraslow signal itself is important. Previous work by the authors of this study on infraslow LFP amplitudes have strongly supported this hypothesis (Pan et al., 2013). Pan et al. and the present study, when combined, suggest that the infraslow component of the LFP needs to be considered as a biomarker, perhaps of another neural process, rather than something fully co-modulated with fluctuations in high frequency power.

These results suggest that, while both infraslow frequency LFP and higher frequency band-limited power LFP are linked to changes in the resting state fMRI signal, they are not simply arising from a single source. In particular, a lack of phase-amplitude coupling between specific infraslow and specific higher frequency bands was seen, either inconsistent coupling (for dexmedetomidine) or widespread, general coupling due to the burst state (for isoflurane). Additionally, a partial correlation analysis only shows the LFP signals from one frequency band influencing another band's correlation with fMRI in one out of four cases (Figure 6, Data sheet 7), and this case was under isoflurane where the burst state is likely to have influenced results. Therefore, either multiple neural influences to the resting state signal must exist (observable as different frequency bands affecting different parts of the fMRI signal), or, if it is a single source, it has a complex relationship between parts of the dynamic resting state signal and which frequency bands of LFP are currently related to it.

### Previous studies of infraslow phase vs. high frequency amplitude coupling

In a 2011 review, Raichle hypothesized that the infraslow fluctuations in resting state fMRI may emerge from phase-amplitude coupling between the infraslow band used in resting state fMRI studies and higher frequency neural electrical activity (Raichle, 2011). Raichle suggests that infraslow potential changes under 1 Hz may reflect changes in cortical excitability, and thus reflect the same type of activity as seen in power changes in higher frequency bands.

Prior to the present study, evidence that infraslow potentials may be linked to faster activity through phase-amplitude coupling was limited, but strong. Vanhatalo et al. used non-invasive human EEG and compared phases from 0.01 to 0.2 Hz to amplitudes >0.5 Hz (Vanhatalo et al., 2004). They observed in both normal subjects and subjects with epilepsy a coupling between the trough of the infraslow wave's phase and high amplitudes in higher frequency activity, and that subjects with epilepsy had epileptic events locked to the infraslow phase as well. Monto et al. investigated the relationship between the 0.01 to 0.1 Hz phase and 1.25 to 40 Hz amplitudes in healthy human subjects who were performing a somatosensory stimulus detection task (Monto et al., 2008). They observed that high power in every high frequency band was linked to the ascending part of the infraslow wave's phase, and that hit rate was similarly phase-locked. Monto et al.'s and Vanhatalo et al.'s results both support the hypothesis put forth by Raichle (2011) that the infraslow potentials reflect a variation in cortical excitability. Further evidence supporting this idea includes the observation that transcranial magnetic stimulation's effectiveness varies with the presence of infraslow activity (Bergmann et al., 2012), and models that assume infraslow activity arises from amplitude fluctuations in higher frequency activity create similar coupling between infraslow activity and hit rate as Monto et al. saw (Lundqvist et al., 2013). This was not seen in the present study where no consistent coupling is seen under dexmedetomidine, and the coupling under isoflurane appears to be due to neural suppression.

The results of the present study, however, do not well support Raichle's hypothesis of variation in cortical excitability driving the infraslow BOLD signal (Raichle, 2011). Notably, no significant phase-amplitude coupling was seen between high frequency LFP power and BOLD phases (Figure 3, bottom row). This does not appear to be merely sub-threshold significance, as when individual histograms are examined, no consistent trend is seen either (Data sheet 6). However, this may be due to a limitation in methods; dexmedetomidine may have specific suppression effects on higher frequency activity (Pan et al., 2011b, 2013). Therefore, future work using awake rodent models may help elucidate if Raichle's hypothesis is testable in rodents.

### Multiple scales in dynamic studies of resting state activity

Previous work supports the idea of multiple time scales of dynamics. QPP are seen robustly in the correlation maps between infraslow LFP and fMRI (Pan et al., 2013; Thompson et al., 2013b) yet were not observed in the correlation maps to band-limited power of higher frequencies (Pan et al., 2011b), despite both bands of the LFP correlating with localized fMRI-BOLD. Thus far, sliding window correlation in fMRI has been shown to consistently relate to sliding window correlation in electrophysiological band-limited power (Chang et al., 2013; Thompson et al., 2013a), but has not yet been linked to infraslow potentials. While much further work is needed, the implication of these early studies is that more localized dynamics (such as sliding window correlation between contralateral homologs) is more closely linked to higher frequency activity, while broader patterns (such as QPP) are more closely linked to infraslow activity.

Future work investigating the neural basis of dynamics in resting state fMRI should take the multiple frequency scales into consideration. For example, if two areas interact with a range of different states represented by different levels of correlation (Keilholz et al., 2013), the states may be visible in a high frequency but a slower, larger scale pattern may either (1) modulate which state is present or, as suggested by the results of this study, (2) be non-correlated with the state. The second case presents a potential problem, as the dynamic state of correlation may be key to understanding the neural basis of functional connectivity (Hutchison et al., 2013a,b; Keilholz et al., 2013) and if the present state is primarily represented in a narrow frequency band, it may be difficult to detect without knowing where to find it. While resting state fMRI studies have long regressed so-called nuisance signals such as the whole brain signal (Fox et al., 2009), a more sophisticated form of regression may be necessary to separate different dynamic components of resting state fMRI that may be both arising from neural sources, but uncorrelated.

### Technical limitations

The power spectra for the high frequencies used in this study show a steady decay as frequency increases, indicating the 1/f drop-off or “pink noise” characteristic of natural signals (Figure 2). This reduction in power at higher frequencies may limit the results possible from comparing power in these frequencies to the fMRI signal, as is seen in Figure 6 where, despite significant correlation, *z*-scores are up to 30 times smaller than those seen with infraslow data. All data used in this study were recorded with no high-pass filter so as to simultaneously record the infraslow and higher band signal, but future work may improve results by splitting the signal to two amplifiers, one optimized for infraslow recording, and the other optimized for 1–100 Hz recording.

In Figure 3, the bench data loses statistical significance for the comodulogram at the higher end of the low frequencies (~0.7 Hz), but these frequencies are significant for scanner data. This may be because the scanner data was recorded in an electromagnetically shielded room, so was able to preserve a broader band of infraslow frequencies. Conversely, the lack of scanner noise present in the power spectra (Figure 2) may improve the bench data, and thus it is not as susceptible to the burst state induced phase-amplitude coupling under isoflurane as the scanner data was.

Due to the long computation time of mean comodulograms (~12 h each), only a single shuffled data set was used, and compared to actual data with a student's *T*-test or a *KS*-test. The use of only a single shuffled dataset unfortunately limited statistical power which caused an increase in both Type I (false positive) and Type II (false negative) errors. Type I errors are a concern, but were vetted against in this study by performing multiple comparisons correction (Section Significance Testing) and by examining individual comodulograms for consistency (Section Consistency of Phase-Amplitude Coupling). As this study's conclusion was, in general, a lack of consistent phase-amplitude coupling, Type I errors are unlikely to have affected it (as they would have resulted in additional, spurious phase-amplitude coupling). Type II errors may be a greater concern, however, as these methods may not find borderline significant results. This is why several representative individual cases were examined (Figure 5, Data sheet 6): to determine if the averages tested obscured non-significant, yet still visible, trends in the data. If computation time can be eliminated as an issue, a better method may be to generate a large number of shuffled data sets and calculate *p*-values based on the distribution of the shuffled data instead of using *T*-tests or *KS*-tests. Another limitation imposed by long computation times was the use of a hard-edged Fourier filter which could be computed more quickly (several orders of magnitude faster) in *MATLAB*. Some of the spurious significance, such as was detected under dexmedetomidine, could potentially be eliminated by a soft-edged filter to reduce dramatic frequency changes and thus edge artifacts (Kramer et al., 2008).

Finally, a major limitation is that the rats were imaged under anesthesia, rather than being awake but quiescent as in most human studies. The use of two different anesthesia, however, provides validation of which results are anesthesia-specific; see appendix A of Thompson et al., 2013b (Thompson et al., 2013b) for further discussion. Despite this, some observations may be due to the anesthetized state in general. Therefore, studies in awake rodents would provide an important link between the anesthetized rodent work and awake human studies.

### Conclusion

The results seen in this study, that phase-amplitude coupling does not largely exist in rats under dexmedetomidine and its existence under isoflurane is likely due to the burst state, is in direct contrast to its motivating hypothesis: that results comparing the infraslow LFP (Thompson et al., 2013b) and higher frequency band-limited power from the LFP (Thompson et al., 2013a) to fMRI may have shared a common mechanism. Instead, it appears that they likely result from different underlying sources that may be neural, glial, or hemodynamic. Previous research in phase-amplitude coupling using infraslow frequencies has been limited, and thus does not preclude our results, but our results do not generally support the overall hypothesis that infraslow oscillations drive higher frequency oscillations (Hughes et al., 2011). One potential commonality is the possibility that the coordinated bursting under isoflurane matches coordinated slow waves in normal sleep in humans, as both produced similar histograms for amplitude vs. phase (Figure 4 in this study, Figure 2 in Vanhatalo et al., 2004).

As the different scales of neural activity that correlate with fMRI do not emerge through nested frequencies, and the lack of linear influence between frequencies (as shown by lack of shared variance in partial correlation, see Figure 6, right side), indicates that multiple scales of dynamics may need to be investigated to fully understand resting state functional networks. Thus, the results of this study should direct researchers to investigate multiple frequency scales when looking at the dynamics in resting state fMRI, and potentially multiple spatial scales as well. For researchers attempting to understand the neural basis of the dynamics in resting state fMRI, this study's results suggest they must include a range of LFP frequencies: merely investigating 1–100 Hz will not, for example, necessarily provide a surrogate result for infraslow potentials.

It has been suggested that investigation of the dynamics of resting state fMRI “may provide greater insight into fundamental properties of brain networks” (page 1, Hutchison et al., 2013a). However, to avoid what seem to be contradictions in such insights as the authors of this study have previously encountered (compare Thompson et al., 2013a vs. Thompson et al., 2013b), it is necessary to understand that there may be multiple processes at work. While not displaying a clear picture of nested frequencies as expected, the present study has shown initial evidence that there may be several underlying processes making up the dynamics seen in resting state fMRI, and separation of them may better allow scientists to tap into the promised potential of dynamic resting state fMRI.

## Funding

NIH, 1R21NS072810-01A1, 1R21NS057718-01A2, and 1R01NS078095-01A1; Scholarly Inquiry and Research at Emory (SIRE) Fellowship program at Emory University.

### Conflict of interest statement

The authors declare that the research was conducted in the absence of any commercial or financial relationships that could be construed as a potential conflict of interest.
