# Supplementary material for: Phase-amplitude coupling and infraslow (<1 Hz) frequencies in the rat brain: relationship to resting state fMRI
Source: Front Integr Neurosci. 2014 May 27;8:41. doi: 10.3389/fnint.2014.00041 (PMC4034045; doi:10.3389/fnint.2014.00041)
Supplement: Supplementary file 1 [file DataSheet1.DOCX]

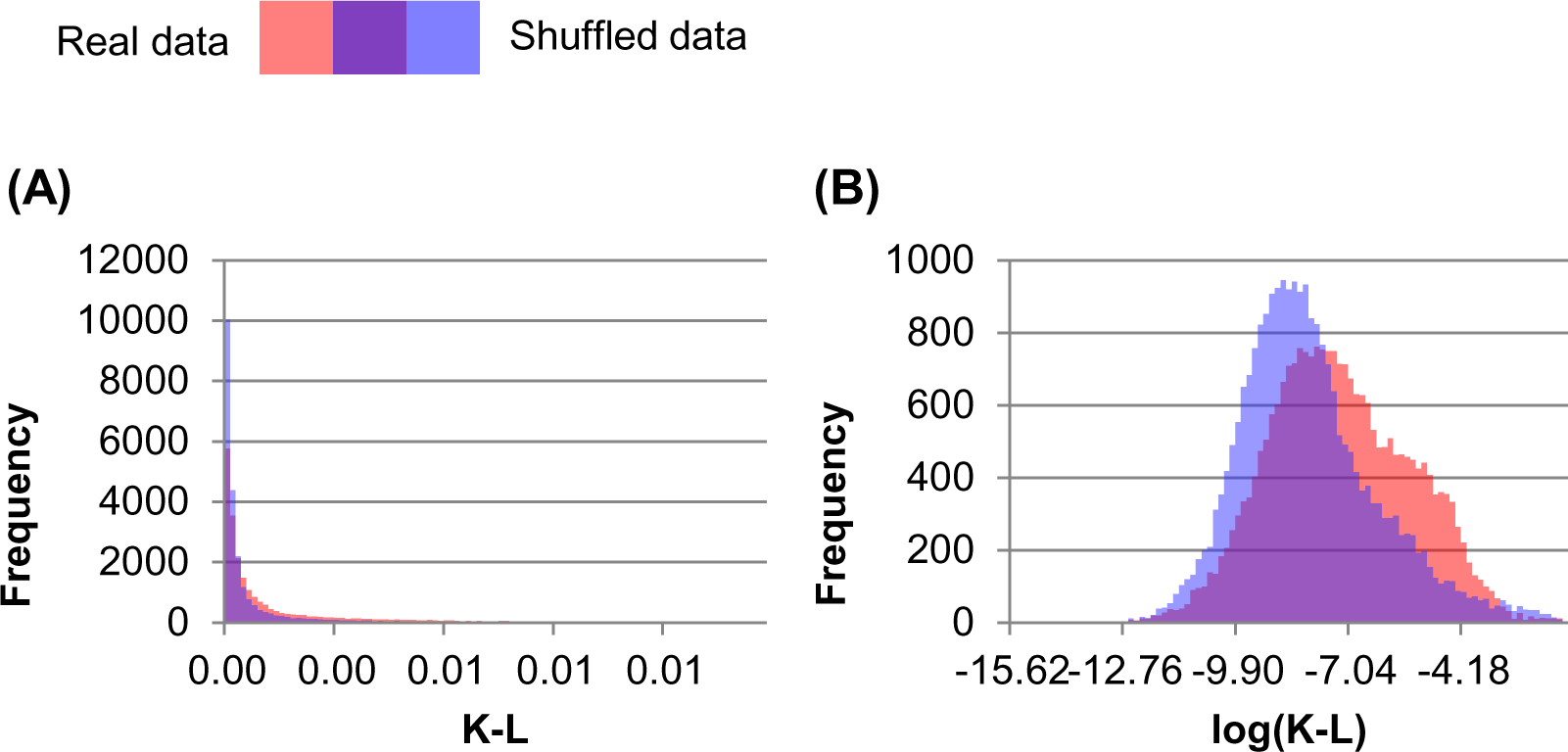


# Data sheet 1

Example of normalization process prior to statistical testing. **(A)** Histogram of K-L values resulting from bench-recorded isoflurane data across all scans and all points on each scan’s comodulogram. The lower 99 bins of 1,250 bins are shown (for comparison, as most bins are empty). Actual data are shown in red, shuffled data in blue, overlap in purple. While there is a bias for actual data being higher values, both distributions show exponential decay. **(B)** Histogram of natural logarithm of K-L values from (A). The lower 99 bins of 100 bins are shown. Data now more closely resemble a normal distribution, the bias in actual data is more apparent, and can be tested with a student’s T-test.
