# Supplementary material for: Phase-amplitude coupling and infraslow (<1 Hz) frequencies in the rat brain: relationship to resting state fMRI
Source: Front Integr Neurosci. 2014 May 27;8:41. doi: 10.3389/fnint.2014.00041 (PMC4034045; doi:10.3389/fnint.2014.00041)
Supplement: Supplementary file 3 [file DataSheet3.DOCX]

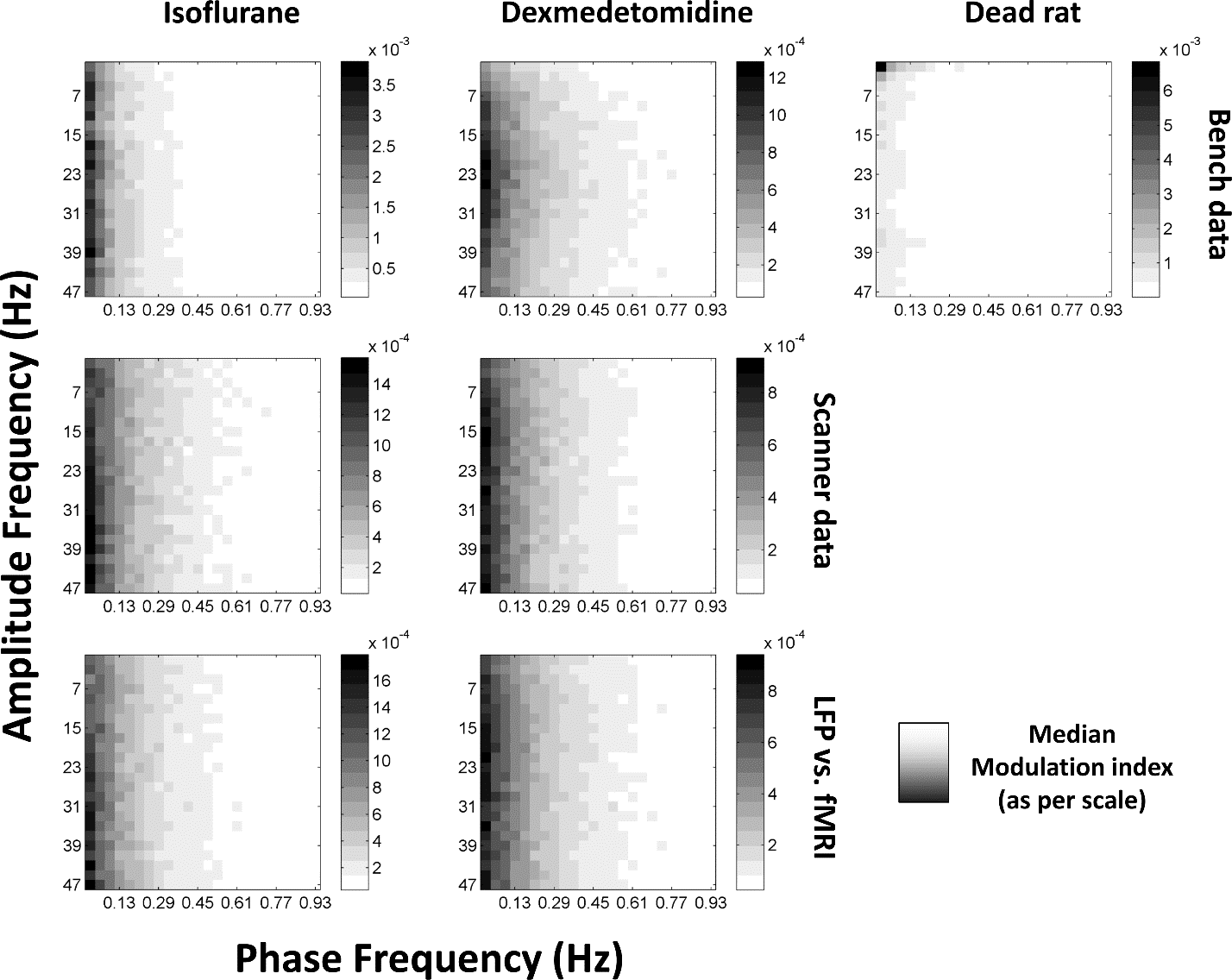


# Data sheet 3

Median comodulograms for each condition with shuffled data. Amplitude frequencies are plotted against phase frequencies with darker values representing greater phase-amplitude coupling. Each plot is scaled between minimum and maximum modulation index, with scale shown on the right. Top row represents data recorded outside the fMRI scanner, middle row data inside the scanner during simultaneous imaging. Bottom row is the same as the middle row but has had the phase signal replaced with equivalent simultaneous fMRI data. Left column is isoflurane anesthesia (N=21 for bench, 17 for scanner, times 2 electrodes), middle column is dexmedetomidine (N = 20 for bench, 39 for scanner, times 2 electrodes) anesthesia, right column is following euthanatization of the rat (N = 10 times 2 electrodes, only available outside the fMRI scanner).
