# Supplementary material for: Phase-amplitude coupling and infraslow (<1 Hz) frequencies in the rat brain: relationship to resting state fMRI
Source: Front Integr Neurosci. 2014 May 27;8:41. doi: 10.3389/fnint.2014.00041 (PMC4034045; doi:10.3389/fnint.2014.00041)
Supplement: Supplementary file 4 [file DataSheet4.DOCX]

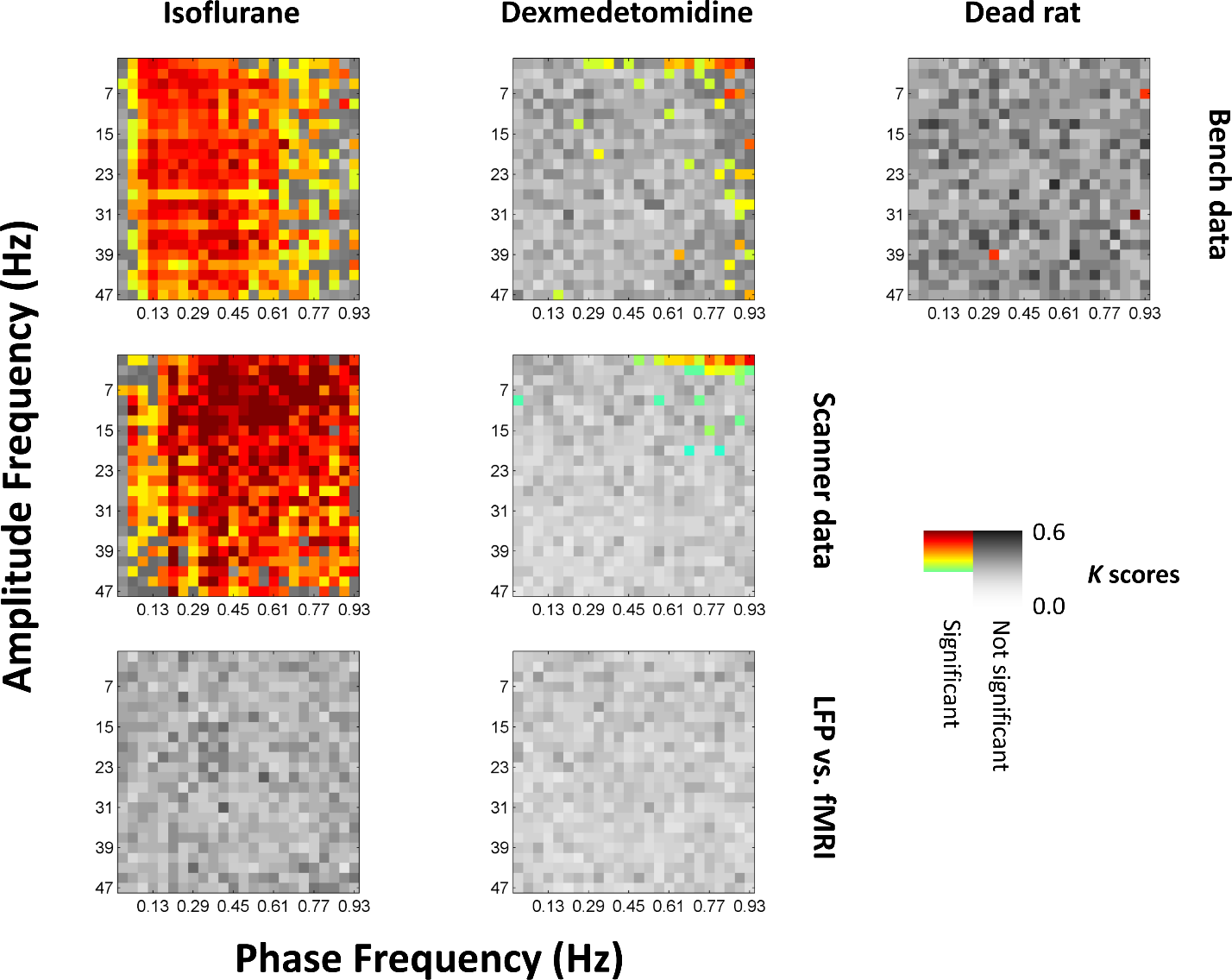


# Data sheet 4

Identical to figure 3 in the main text, except a *KS* test was used to determine significance instead of a *t* test, and the colors indicate *K* scores resulting from that test rather than *t* scores. Results are similar under both types of test.
