# Supplementary material for: Phase-amplitude coupling and infraslow (<1 Hz) frequencies in the rat brain: relationship to resting state fMRI
Source: Front Integr Neurosci. 2014 May 27;8:41. doi: 10.3389/fnint.2014.00041 (PMC4034045; doi:10.3389/fnint.2014.00041)
Supplement: Supplementary file 5 [file DataSheet5.DOCX]

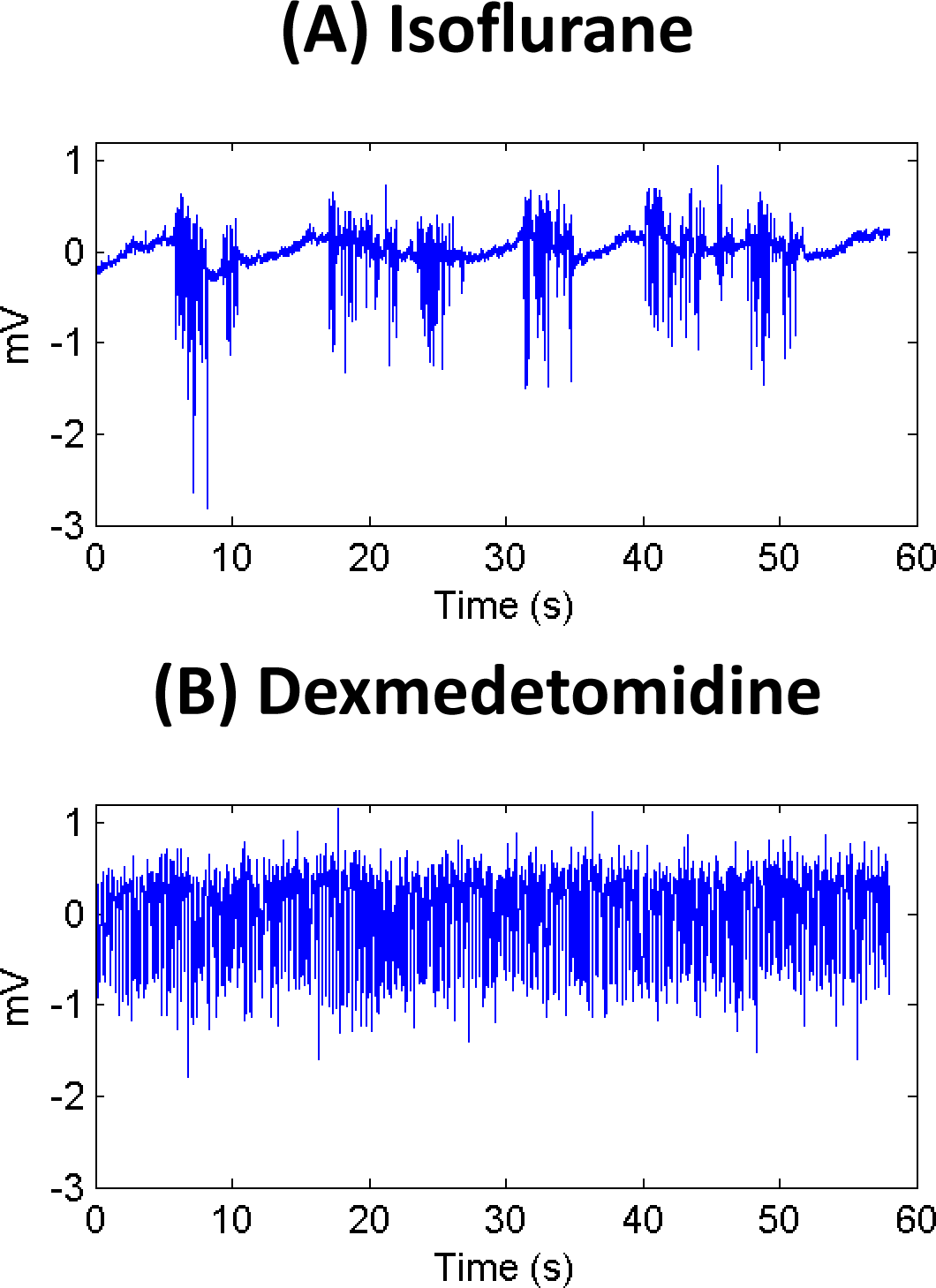


# Data sheet 5

Example LFP traces under each anesthetic, **(A)** isoflurane and **(B)** dexmedetomidine. Note that activity is fairly constant under dexmedetomidine, while isoflurane alternates between periods of neural suppression and periods of activity. This is referred to as a “burst state.”
