# Supplementary material for: Phase-amplitude coupling and infraslow (<1 Hz) frequencies in the rat brain: relationship to resting state fMRI
Source: Front Integr Neurosci. 2014 May 27;8:41. doi: 10.3389/fnint.2014.00041 (PMC4034045; doi:10.3389/fnint.2014.00041)
Supplement: Supplementary file 6 [file DataSheet6.DOCX]

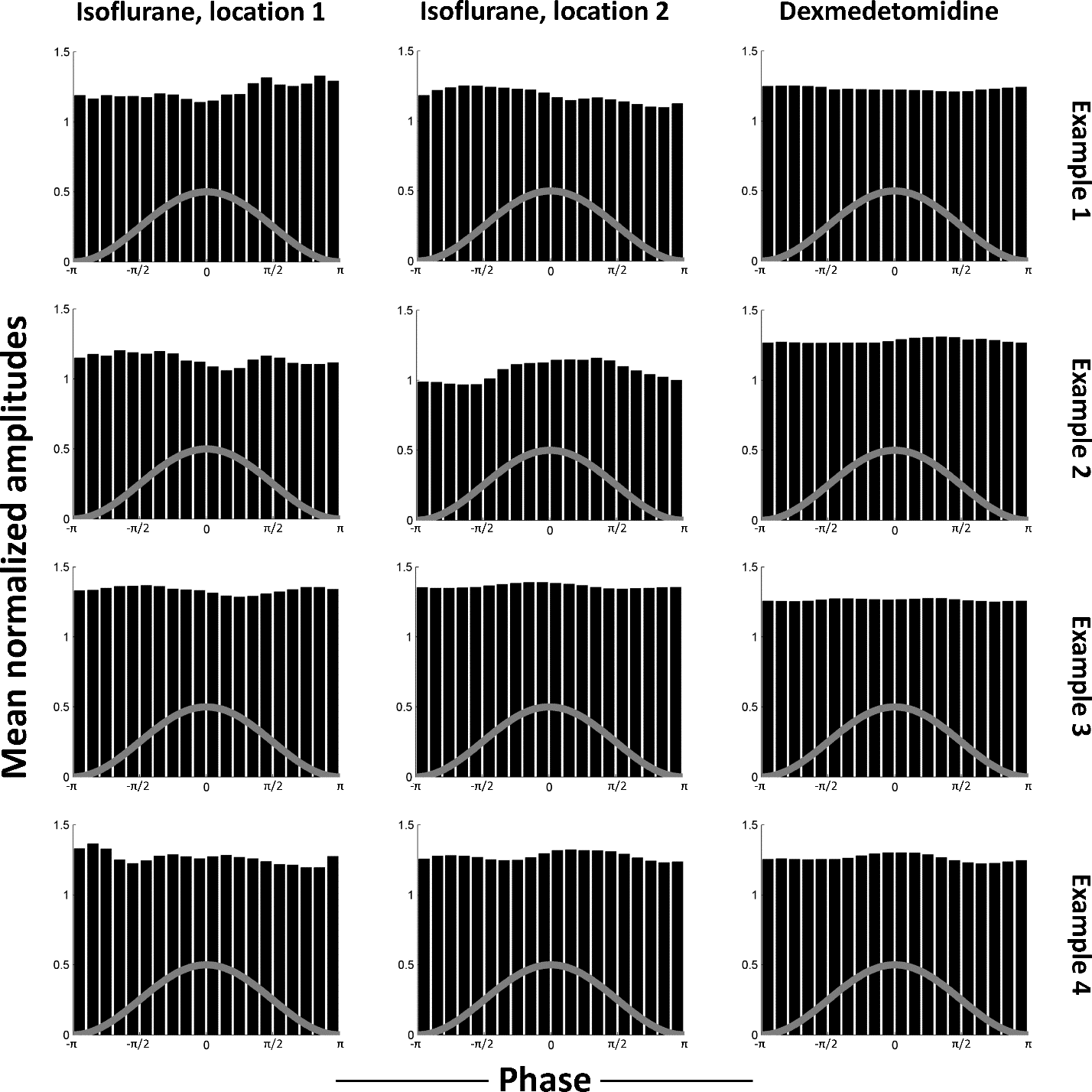


# Data sheet 6

As Figure 5 in the main text, except histograms are shown from scanner data, where high frequency LFP amplitudes were compared to infraslow BOLD phases rather than infraslow LFP phases. Unlike from comparing LFP amplitudes to LFP phases, no clear trend is visible here.
