# Supplementary material for: Phase-amplitude coupling and infraslow (<1 Hz) frequencies in the rat brain: relationship to resting state fMRI
Source: Front Integr Neurosci. 2014 May 27;8:41. doi: 10.3389/fnint.2014.00041 (PMC4034045; doi:10.3389/fnint.2014.00041)
Supplement: Supplementary file 7 [file DataSheet7.DOCX]

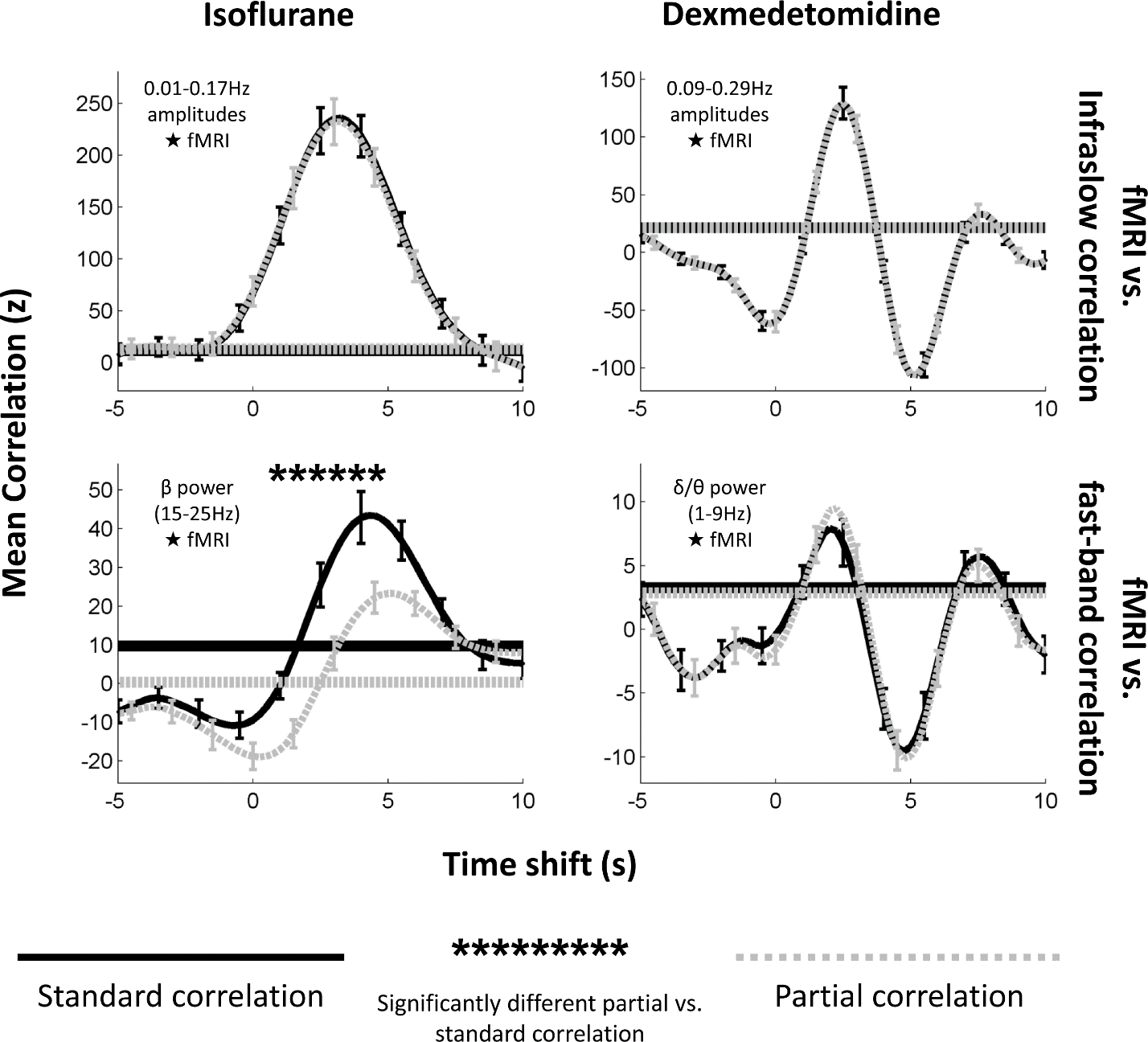


# Data sheet 7

Identical to Figure 6, part B from the main text, except that Spearman correlation coefficients were used instead of Pearson correlation coefficients. Results are similar under both correlation coefficients.
